# Indication of Quantitative Multiple Disease Resistance to Foliar Pathogens in *Pinus radiata D.Don* in New Zealand

**DOI:** 10.3389/fpls.2020.01044

**Published:** 2020-07-10

**Authors:** Ahmed Ismael, Mari Suontama, Jaroslav Klápště, Stuart Kennedy, Natalie Graham, Emily Telfer, Heidi Dungey

**Affiliations:** ^1^ Forest Genetics, Scion, Rotorua, New Zealand; ^2^ Tree Breeding, Skogforsk, Umeå, Sweden

**Keywords:** needle retention, foliar diseases, heritability, genetic correlation, disease resistance, *Dothistroma*, *Cyclaneusma*, *Phytopththora pluvialis*

## Abstract

Increasing resistance against foliar diseases is an important goal in the *Pinus radiata* D.Don breeding program in New Zealand, and screening for resistance has been in place for some time, since the late 1960s. The current study presents results of four progeny trials within the breeding program to investigate whether multiple disease resistance could be detected against three different needle diseases in *P. radiata*: *Dothistroma* needle blight (DNB) caused by *Dothistroma septosporum*, *Cyclaneusma* needle cast (CNC) caused by *Cyclaneusma minus*, and red needle cast (RNC) caused by *Phytophthora pluvialis.* Four progeny trials in the North Island of New Zealand were available to estimate heritabilities and between-trait genetic correlations. Two of the trials were assessed for DNB, involving 63 full-sib families. A third trial was assessed for CNC, involving 172 half-sib families, and a fourth trial was assessed for RNC, involving 170 half-sib families. Disease resistances had moderate estimates of heritability (0.28–0.48) in all trials. We investigated the potential for multiple disease resistance to the three foliar diseases by estimating genetic correlations between disease resistances using a spatial linear mixed model. The correlation between DNB and CNC resistance was favorable and strong (0.81), indicating that genotypes that are highly resistant to DNB also have a high resistance to CNC. These results suggest that selection based on resistance to DNB could allow for simultaneous indirect selection for resistance to CNC, usually only expressed at a later age. This would allow selections to be made earlier due to the earlier expression of DNB than CNC and reduce the number of expensive disease assessments being undertaken. Conversely, genetic correlation estimates for RNC with DNB and CNC were close to zero, and very imprecise. As such, later-age assessments for this disease would still be required.

## Introduction

*Pinus radiata* D. Don comprises 90% of the 1.7 million hectares of exotic forest plantation area in New Zealand (NZFOA, 2019). *Pinus radiata* plantations in New Zealand do, however, face serious threats due to foliar diseases (Burdon, 2008). These include *Dothistroma* needle blight (DNB), *Cyclaneusma* needle cast (CNC), red needle cast (RNC), and physiological needle blight (PNB). The cost of resulting growth and productivity losses from these needle diseases to New Zealand is significant. For example, a 10% increase in the proportion of *P. radiata* trees affected by CNC equated to a loss in volume of 10–14 m^3^ ha^−1^ per annum (Van Der Pas et al., 1984b). A similar loss in volume of 7–13 m^3^ ha^−1^ was estimated per 10% increase in the proportion of DNB affected trees (Van Der Pas, 1981). To put these values into context, the economic cost of DNB to the New Zealand forest industry has been estimated at $19.8 million (NZD) per annum from lost productivity and the cost of chemical control measures such as aerial spraying of copper oxides (Watt et al., 2011a). The economic cost of CNC is likely even higher, with some estimates of $38 million (NZD) per annum for trees aged between 6 and 20 years (Bulman and Gadgil, 2001; Watt et al., 2012).

DNB is a needle disease that has been identified from 76 countries, infecting 109 documented host species across their native and non-native ranges (Bednářová et al., 2006; Barnes et al., 2014; Drenkhan et al., 2016). The fungus causing the disease, *Dothistroma septosporum* (Dorog.) Morelet, is under the Ascomycota division in the Fungi kingdom. In New Zealand, DNB was first observed in in 1962 in young *P. radiata* plantations (Bulman et al., 2004). The pathogen is suggested to have been introduced by foresters who had visited East Africa in 1957 to study DNB (Hirst et al., 1999; Barnes et al., 2014). By the end of the 1960s, the disease spread across most of the North Island of New Zealand and had spread across the South Island by the late 1990s (Bulman et al., 2004; Bulman et al., 2013; Bulman and Gardner, 2014). DNB occurs in young trees up to approximately 15 years of age, with needle symptoms usually first appearing toward the end of summer and becoming most apparent in winter (Bulman et al., 2004), however, infection is usually present all year round. The disease is characterized by a distinctive brick-red banding pattern (1–3 mm wide) on the needles, resulting from the production of the fungal toxin, dothistromin (Shain and Franich, 1981; Kabir et al., 2015). Stromata, small black fruiting bodies, appear within these red bands in later stages of the disease, from which conidia, the asexual spores, are released and dispersed by water splash from tree to tree over a relatively small distance (Gadgil, 1967; Ivory, 1972). Small water droplets, 50 µm in diameter (i.e., mist or fogs), may contain *D. septosporum* conidia that can be carried by wind, and travel considerable distances (Gibson et al., 1964; Mullett M.S. et al., 2016). Furthermore, *D. septosporum* may continue to release spores from fruiting bodies in dead needles on the forest floor, where viable conidia can survive for up to 32 weeks (Gadgil, 1970; Mullett M. et al., 2016). DNB is widespread in warm, moist conditions, as found within the central North Island of New Zealand (Watt et al., 2011b). Breeding for DNB resistance in New Zealand and Australia has shown some success, perhaps aided by low pathogen diversity due to the absence of the sexual form in these countries (Hirst et al., 1999).

CNC has been reported in more than 45 countries, and is known to infect approximately 37 pine species and subspecies (CABI, 2011; Farr and Rossman, 2012). Pathogenicity, however, has only been demonstrated with *P. radiata* and *P. sylvestris* (Gadgil, 1984; Merrill and Wenner, 1996), with *Cyclaneusma minus* first documented on *P. radiata* in New Zealand in the 1950s (Bulman and Gadgil, 2001). The fungus causing the disease, *Cyclaneusma minus* (Butin) DiCosmo, Peredo, and Minter, is also under the Ascomycota division. CNC typically affects needles of host trees that are at least 5−6 years of age, but infections are most evident on trees aged 11−20 years. (Van Der Pas et al., 1984a; Bulman and Gadgil, 2001). Symptoms caused by CNC are usually most severe in spring, with less severe in autumn (Bulman and Gadgil, 2001). CNC is characterized by premature casting of mottled yellow-brown needles, usually 1-year-old or older in age. Fruiting bodies are produced on both attached, living needles as well as the cast needles lying on the forest floor, mostly in autumn-winter (May to August), with spores dispersed to new host trees by wind. As with DNB, CNC is widespread in the warm moist conditions found within the central North Island of New Zealand (Watt et al., 2012).

In 2008, a new needle disease that caused defoliation of *P. radiata* was found on the east coast of the North Island of New Zealand, near Gisborne (Dick et al., 2014). Named RNC, the disease is characterized by discrete khaki-colored bands that appear on infected needles, which then turn red and are prematurely cast (Dick et al., 2014). This disease is caused by the fungal-like oomycete *Phytophthora pluvialis* Reeser, Sutton & E Hansen, which falls under the division Heterokontophyta. It tends to primarily affect the older needles, while the new flush remains healthy thus preventing complete defoliation of affected trees (Ganley et al., 2014). Symptoms may start any time between summer and winter in trees aged 3 or older, but symptoms are usually most severe from winter onward, depending on the region within New Zealand (Bulman and Gardner, 2014). However, the topographic distribution of infection is different from other needle diseases: DNB and CNC generally infect foliage most heavily in sheltered gullies and hollows, while RNC infection tends to be more prominent on ridgetops where needles are exposed to continual wet and misty conditions (Dungey et al., 2014). While reports of the disease have been widespread across different parts of New Zealand, outbreaks within breeding trials have been limited (Ganley et al., 2014). For that reason, it was difficult to quantify the productivity loss associated with RNC. One study, however, reported 35% growth loss in the year following severe disease, and an average growth reduction of 16% over a 3-year period (P.N. Beets, Scion, unpublished data). In many sites or years, the incidence and severity of RNC is not high enough to cause any significant reduction in tree growth (Ganley et al., 2014).

PNB has been causing severe episodes of defoliation in number of locations throughout New Zealand since the early 1980s (Bulman et al., 2008). This disease is characterized by the appearance of red-brown discoloration on the affected needles, which then turn entirely red-brown and are eventually cast. Unlike CNC or RNC, however, the dead needles usually remain attached to the branches (i.e., unlike CNC or RNC) (Bulman and Gardner, 2014). The disease tends to primarily affect trees over the age of 12 years but will also affects younger trees where it is easily confused with DNB (Dick and Bulman, 2004). Once believed to be caused by an unidentified fungus, inconsistent isolations of fungal species from diseased needles led to the conclusion that it was abiotic in origin (Dick and Bulman, 2004). However, evidence is now building that supports the involvement of *Phytophthora* species in this disease (S. Fraser, Scion, unpublished data). Symptoms of PNB may start at winter, but are usually most severe in September-November (Bulman and Gardner, 2014). While symptoms might occur throughout the stand, trees growing in gullies are usually most severely affected, likely due to the continual exposure to wet conditions (Bulman and Gardner, 2014).

Tree resistance against biotic needle diseases has been found to be under moderate genetic control, indicating that selection for these traits could help breeders improve disease resistance. For example, heritability for DNB had a median of 0.36 in 16 trials of 3 and 4 year old *P. radiata* in Australia (Ivković et al., 2010), and estimated heritability of CNC ranged from 0.25 to 0.46 in three progeny trials of *P. radiata* in New Zealand and Australia (Suontama et al., 2019). Selection for CNC resistance is, however, less straightforward, as infection does not usually become obvious in stands less than 6 years of age (Bulman and Gadgil, 2001), at which point it becomes more difficult to assess due to the height of tree crowns and canopy closure. Furthermore, the assessment of one disease is often confounded by the earlier or concomitant presence of one or more other foliar diseases.

Selection for resistance to multiple diseases, either through direct selection for the main disease trait, or indirect selection using correlated traits, may be possible if adequate genetic correlation exists between resistance traits. Multiple disease resistance arises when the same set of genes control the response to more than one disease (Wisser et al., 2005; Chung et al., 2010), however, there are not many reports of this phenomena in plants. In one example, Wisser et al. (2011) estimated the genetic parameters for disease resistances among northern leaf blight (*Exserohilum turcicum*), gray leaf spot (*Cercospora zeae-maydis*), and southern leaf blight (*Cochliobolus heterostrophu*) for 250 maize lines, with estimated genetic intercorrelations ranging from 0.55 to 0.67. The fact that the genetic correlations were significantly different from zero suggested that resistance to multiple diseases is possible. In forest tree species, breeding programs in American chestnut are seeking to improve resistance to chestnut blight (*Cryphonectria parasitica*) and to phytophthora dieback (*Phytophthora cinnamomi*) through backcrossing between American and Chinese chestnut (Burnham et al., 1986). However, no evidence of multiple disease resistance has been found to chestnut blight and phytophthora dieback in the first-year American chestnut and Chinese chestnut hybrids (Westbrook et al., 2019). To our knowledge, there have been no previous studies investigating multiple disease resistance in pine species. Multiple disease resistance, if it exists, would not only simplify selection processes, but earlier indirect selections might also be possible for diseases that are usually only expressed in older trees, with the ultimate aim of targeting multiple disease resistant selections to locations where multiple diseases are present.

Precise needle diseases assessments for genetic evaluation purposes depend heavily on the accurate identification of the disease in the field. Key diagnostics features of the four common needle diseases in New Zealand (i.e., DNB, CNC, RNC, and PNB) have been described by Bulman and Gardner (2014). Using molecular tools to confirm the causative agent alongside visual assessments would be useful to deliver more precise genetic evaluations for each needle disease. However, this is not usually logistically feasible at the scale required for genetic evaluation purposes due to the large number of trees assessed in each trial.

In the current study, disease was visually assessed in four progeny trials within the *P. radiata* breeding program in New Zealand, each naturally infected with one of three specific needle diseases. Our objectives were (1) to estimate variance components and heritability estimates for resistance against DNB, CNC, and RNC; (2) to investigate whether multiple disease resistance could be detected against the three different pathogens that cause needle damage; (3) to estimate the correlated response to selection based on genetic correlations between traits; and (4) to investigate whether there was any genotype by environment interaction level (G×E) for resistance against DNB in two progeny trial sites.

## Materials and Methods

### Description of Trials

Four progeny trials within the New Zealand Radiata Pine Breeding Company's breeding population were used to estimate the genetic parameters for disease resistances (**Table 1**). All four trials were randomized incomplete block designs with an “optimal design” (Butler, 2013) as a single-tree plot. Each of these trials was assessed for only one disease, as they did not appear to be infected by other diseases at the time of the current study (Bulman and Gardner, 2014). All diseases were naturally occurring and not the result of artificial inoculations. Two of the trials were clonally replicated, derived from 63 pair-cross families, and were established across two sites located in the North Island of New Zealand, namely Kaingaroa and Kinleith forests (Kinleith-D) (**Table 1**). The Kaingaroa forest trial contained 3079 trees in total, from 1379 genotypes, and the Kinleith forest trial contained 3,080 trees in total, from 1,263 genotypes. DNB resistance was assessed in these two trials at two years after planting. The third trial, derived from 172 open-pollinated families (deemed close to approximately half-sib), was established in Kinleith forest (Kinleith-C) and contained 4,500 trees in total. This trial was assessed for CNC resistance 5 years after planting. This assessment was slightly early in terms of when expression of this disease is usually observed, however, the stand was due for pruning which would have compromised the ability to accurately determine needle retention. The fourth trial was derived from 170 open-pollinated families, established in Mahurangi forest, and contained 4,500 trees in total. This trial was assessed for RNC resistance 9 years after planting. There was a total 13,713 trees assessed in all sites.

**Table 1:** Description of progeny trials at Kaingaroa, Kinleith, and Mahurangi forests in New Zealand.

**Trial**	Kaingaroa	^1^Kinleith-D	^2^Kinleith-C	Mahurangi
Disease assessment	*Dothistroma* needle blight		*Cyclaneusma* needle cast	Red needle cast
Trial establishment date	August 2014	August 2014	August 2008	July 2006
Latitude	38° 24' 30''	38 ° 18' 52''	38° 15' 56”	36° 22' 29''
Longitude	176° 33' 53''	176° 00' 51''	176° 04' 30”	174° 32' 54''
Assessment date	October 2016	October 2016	December 2013	November 2015
Average season temperature (°C)	12.8	12.9	14.6	13.5
Average season rainfall (mm)	104.4	103.7	61.8	79.2
No. planted trees	3,079	3,080	4,500	4,500
No. blocks	86	86	125	125
No. trees per block	36	36	36	36
Tree spacing (m)	3.1×3.1	3.1×3.1	4.0×3.0	3.0×3.0
Trial design	Incomplete block design with single-tree plots			

Average temperature and rainfall for the season of assessment kindly provided by National Institute of Water and Atmospheric (NIWA).

^1^Kinleith-D = Kinleith site for Dothistroma needle blight assessment.

^2^Kinleith-C = Kinleith site for Cyclaneusma needle cast assessment.

The pedigree for the study incorporated up to four generations. There was a total of 37 parents shared between all sites. The pedigree file included 10,974 genotypes and was used to build the average numerator relationship matrix for the genetic analysis, considering all the individual genotypes in the pedigree file.

### Definition of Traits

Prior to the field assessments, a pathologist visited each site to confirm which disease was present and ascertain whether symptoms at a given site attributable to one or multiple diseases. This was performed by visual inspection of the needle symptoms in a subset of trees, following the guidelines of Bulman and Gardner (2014). Thereafter, full trial assessments were performed where symptoms were mostly attributed to one disease in each site and when the expression of these symptoms was discernible and easy to evaluate by the assessor.

Needle disease resistance for each of the diseases was scored as follows:

1. Needle damage due to DNB was subjectively scored as a percentage of the total unsuppressed crown volume present on the tree that was diseased, in 5% intervals from 0 to 90% (Kershaw et al., 1982). For example, a score of 40% indicates that 40% of the foliage present is diseased (Bulman et al., 2004).
2. Needle retention to describe resistance to CNC was scored following Wardlaw et al. (2007, unpublished work) as follows: 0—all 1-, 2-, and 3-year-old needles retained; 1—all 1- and 2-year-old needles retained and partial retention of 3-year-old needles; 2—all 1- and 2-year-old needles retained; 3—all 1-year-old needles retained, with partial retention of 2-year-old needles; 4—only 1-year-old needles retained; 5—partial retention of 1-year-old needles; 6—no needles retained.
3. The incidence of RNC was scored as the percentage of the crown defoliated by RNC in 5% intervals from 0 to 100% (Dungey et al., 2014).

### Statistical Analysis

#### Estimation of Variance Components and Genetic Parameters

Genetic analyses were performed with the average information restricted maximum likelihood (REML) algorithm in the ASReml-R v.3 statistical package (Butler et al., 2009). Single-trait analysis was performed to estimate variance components and heritability for each trait separately, whereas multi-trait analysis was performed to estimate genetic correlations between the traits. To meet the normality assumption, the phenotypic data for each trait were square root transformed prior the analysis.

For DNB in Kaingaroa forest (DNB1) and Kinleith forest (DNB2), the following individual tree linear mixed model (Borralho, 1995) in scalar notation was used:

(1) $$y_{jkl}=\mu +B_{j}+g_{k}+gw_{l}+e_{jkl}$$

where, y_jkl_ is the vector of individual tree observations of needle damage caused by DNB1 or DNB2; *µ* is the overall mean; *B*_j_ is the random block effect $∼\text{N}(0,I\sigma_{B}^{2})$, where $\sigma_{B}^{2}$ is the block variance and *I* is the identity matrix; *g_k_* is the random additive genetic effect which was assumed to be normally distributed $∼\text{N}(0,A\sigma_{g}^{2})$, where $\sigma_{g}^{2}$ is the additive genetic variance and *A* is the average numerator relationship matrix; *gw_**l**_* is the non-additive genetic effect of the individual genotype $∼\text{N}(0,I\sigma_{gw}^{2})$, where $\sigma_{gw}^{2}$ is the non-additive genetic variance, and *e_jk__l_* is the random residual.

For CNC and RNC, the following individual tree linear mixed model (Borralho, 1995) was used:

(2) $$y_{ijk}=\mu +R_{i}+B_{j}+g_{k}+e_{ijk}$$

where *y_ijk_* is the observation of the needle damage caused by CNC, or RNC; *µ* is the overall mean; *R_i_* is the random replication effect $∼\text{N}(0,I\sigma_{R}^{2})$, where $\sigma_{R}^{2}$ is the replicate variance and *I* is an identity matrix; *B_j_* is the random block effect nested within the replicates effect $∼\text{N}(0,I\sigma_{B}^{2})$, where $\sigma_{B}^{2}$ is the incomplete block nested within the replication variance and *I* is an identity matrix*; g_k_* is the random additive genetic effect $∼\text{N}(0,A\sigma_{g}^{2})$, where $\sigma_{g}^{2}$ is the additive genetic variance and *A* is the average numerator relationship matrix; and *e_ij__k_* is the random residual.

Preliminary analyses were performed to test the significance of the improvement achieved through using a spatial autoregressive mixed model over a traditional mixed model without the autoregressive term (i.e., non-spatial), using a likelihood ratio test (LRT). The log likelihoods of the two models were compared against the chi-square distribution (Dutkowski et al., 2006).

Spatial mixed models included the first-order autoregressive random error terms on the rows (row) and columns (col) directions. The residual structure (R) was divided into spatially dependent (ξ), and independent (η) error terms as $\text{R}=\sigma_{\xi }^{2}[\text{AR}1\;(p_{col})⊗\mathrm{AR}1\;(p_{row})]+I\sigma_{\eta }^{2}$, where $\sigma_{\xi }^{2}$is the spatial variance, ⊗ is the Kronecker product, and AR1(*p*) represents a first-order autoregressive correlation matrix for rows and columns where *ρ* is the autocorrelation parameter, *I* is the identity matrix and$\sigma_{\eta }^{2}$ is independent residual variance.

Variance component estimates from the single-trait analysis were used to estimate the narrow-sense heritability (*h^2^*) for DNB1 and DNB2 as follows:

(3) $$h^{2}=\frac{\sigma_{g}^{2}}{\sigma_{g}^{2}+\;\sigma_{gw}^{2}\;+\;\sigma_{\text{η}}^{2}}$$

where $\sigma_{g}^{2}$ is the additive genetic variance, $\sigma_{gw}^{2}$ is the non-additive genetic variance, and the independent error term is $\sigma_{\text{η}}^{2}$.

The narrow-sense heritabilities for CNC and RNC were estimated similarly, however, since these are non-clonal open-pollinated families, the term for non-additive genetic components was not included in these heritability estimates.

Because DNB was assessed in clonally replicated trials, the broad-sense heritability (*H^2^*) for DNB1 and DNB2 was also estimated as follows:

(4) $$H^{2}=\frac{\sigma_{g}^{2}+\sigma_{gw}^{2}}{\sigma_{g}^{2}+\sigma_{gw}^{2}\;+\;\sigma_{e}^{2}}$$

Genotype by environment interaction (G × E) for DNB was measured as the departure from 1 of the genetic correlation between disease assessments across different environments (sites). Model 1 was therefore extended to a bivariate model to estimate the genetic correlation between Kaingaroa and Kinleith, with values below 0.8 deemed to indicate the presence of a significant G × E interaction (Robertson, 1959).

Both models 1 and 2 were extended to a multi-trait analysis to estimate the pairwise genetic correlations between the abovementioned traits to investigate whether multiple disease resistance could be detected against the three different pathogens in the current study. The variance-covariance structure for this model were extended to include $\sigma_{g1_{1}g_{2}},\sigma_{g_{1}g_{3}},\;\sigma_{g_{1}g_{4}},\sigma_{g_{2}g_{3}},\sigma_{g_{2}g_{4}}$, and $\sigma_{g_{3}g_{4}}.$ representing the additive genetic covariances between traits.

Genetic correlations $\left(r_{g_{xy}}\right)$ between traits were estimated as:

(6) $$r_{g_{xy}}=\frac{\sigma_{g_{x}g_{y}}}{\sqrt{\sigma_{g_{x}}^{2}\sigma_{g_{y}}^{2}}}$$

where $\sigma_{g_{x}g_{y}}$ is the additive genetic covariance between traits, and $\sigma_{g_{x}}^{2}$ and $\sigma_{gy}^{2}$ are the individual trait additive genetic variances.

The expected correlated responses to indirect selection (Falconer and Mackay, 1996; Mrode, 2014) were used to evaluate the efficiency of selection for a given trait based on the selection for a genetically correlated trait as *CR_y_* = *i.h_x_.h_y_.r_gxy_.σ_y_*, where *CR_y_* is the correlated response in trait *y* as a result of direct selection on trait *x*, *i* is the intensity of selection for both traits (we assumed equal selection intensities), *h_x_* and *h_y_* are the square root of the heritabilities for the traits, *r_gxy_* is the genetic correlation between the traits, and *σ_y_* is the phenotypic standard deviation for trait (*y*).

Approximate standard errors of estimates of the heritability for each trait and genetic correlations between traits were calculated from the standard errors of the covariance components using a Taylor series expansion approximation, implemented in the ASReml-R package (Butler et al., 2009).

## Results

### Descriptive Statistics

**Figure 1:**
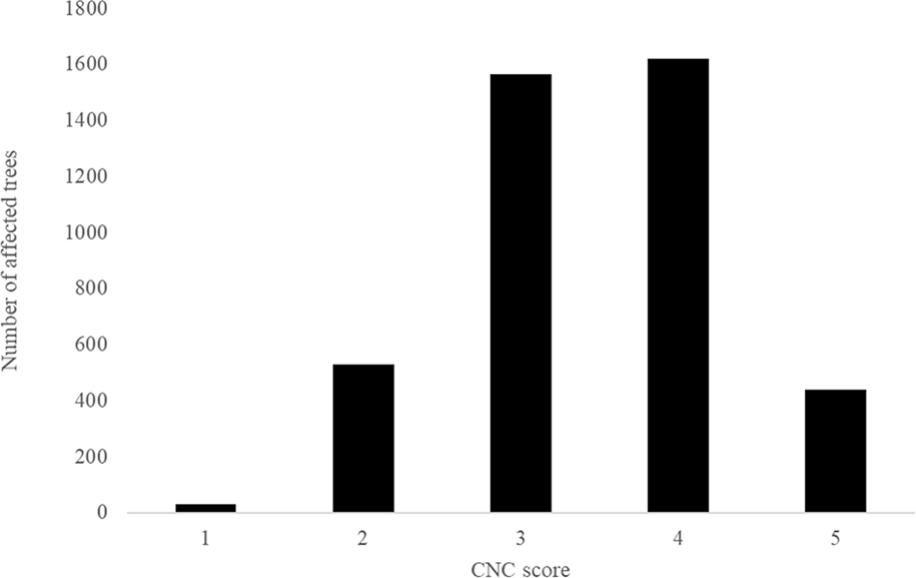
Histogram of *Cyclaneusma* needle cast damage in Kinleith-C (CNC). Smaller scores indicate low disease expression and larger scores indicate high disease expression.

Numbers of planted trees per site, overall means for each disease trait, as well as minima, maxima, individual-tree standard deviations, and percentage of infected trees have been summarized in **Table 2**. The severities of infection were highest for RNC with a mean disease score of 41.83%, and the lowest for DNB1 with mean disease score of 21.14%. Disease incidence for DNB1 and DNB2, expressed as the percentage of trees affected by disease, were 93.4% and 93.1%, respectively. For RNC, the disease incidence was 82.9%. For CNC, 100% of trees appeared to be infected, with a mean retention score of 2.55. While the CNC assessments used quite broad categories, non-normality was not severe (**Figure 1**). Moderate defoliation was observed for most trees in the trial, which retained all 1-year-old needles but were partially or fully defoliated for 2- and 3-year-old needles (i.e., 3,188 trees with score-3 and score-4). Mild defoliation was observed for 566 trees, which retained all 1- and 2- year-old needles but were partially or fully defoliated for 3-year-old needles (i.e., score-1 and score-2). Severe defoliation was observed for 441 trees, with only partially retained 1-year-old needles (i.e., score-5).

**Table 2:** Descriptive statistics for *Dothistroma* needle blight (expressed as percentage crown dieback from 0 to 90%), *Cyclaneusma* needle cast (severity of infection described qualitatively from 1 to 5, with 1 being least and 5 being most infected), and red needle cast disease assessments (expressed as percentage crown affected from 0 to 100%).

Trait	^1^DNB1	^2^DNB2	^3^CNC	^4^RNC
Average infection score	21.14	29.35	2.55	41.83
SD	14.56	17.46	0.87	16.94
Min	0	0	1	0
Max	80	90	5	100
CV%	68.87	59.49	34.12	40.5
Min infected trees per block	27	23	––––	21
Max infected trees per block	36	36	––––	36
Total number of infected trees	2,876 (93.4%)	2,868 (93.1%)	4,500 (100%)	3732 (82.9%)

1 DNB1: Dothistroma needle blight in Kaingaroa forest.

2 DNB2: Dothistroma needle blight in Kinleith forest.

3 CNC: Cyclaneusma needle cast.

4 RNC: Red needle cast.

### Variance Components and Heritability Estimates

**Figure 2:**
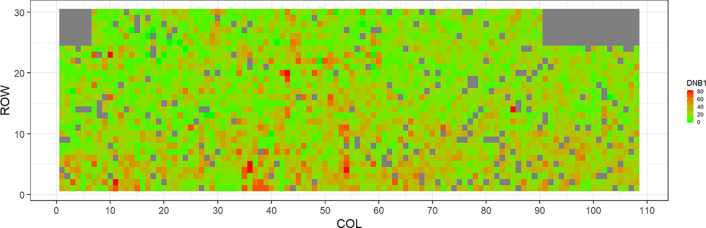
Spatial distribution of *Dothistroma* needle blight damage in Kaingaroa forest (DNB1). Smaller scores indicate low disease expression (greener color) and larger scores indicate high disease expression (redder color).

**Figure 3:**
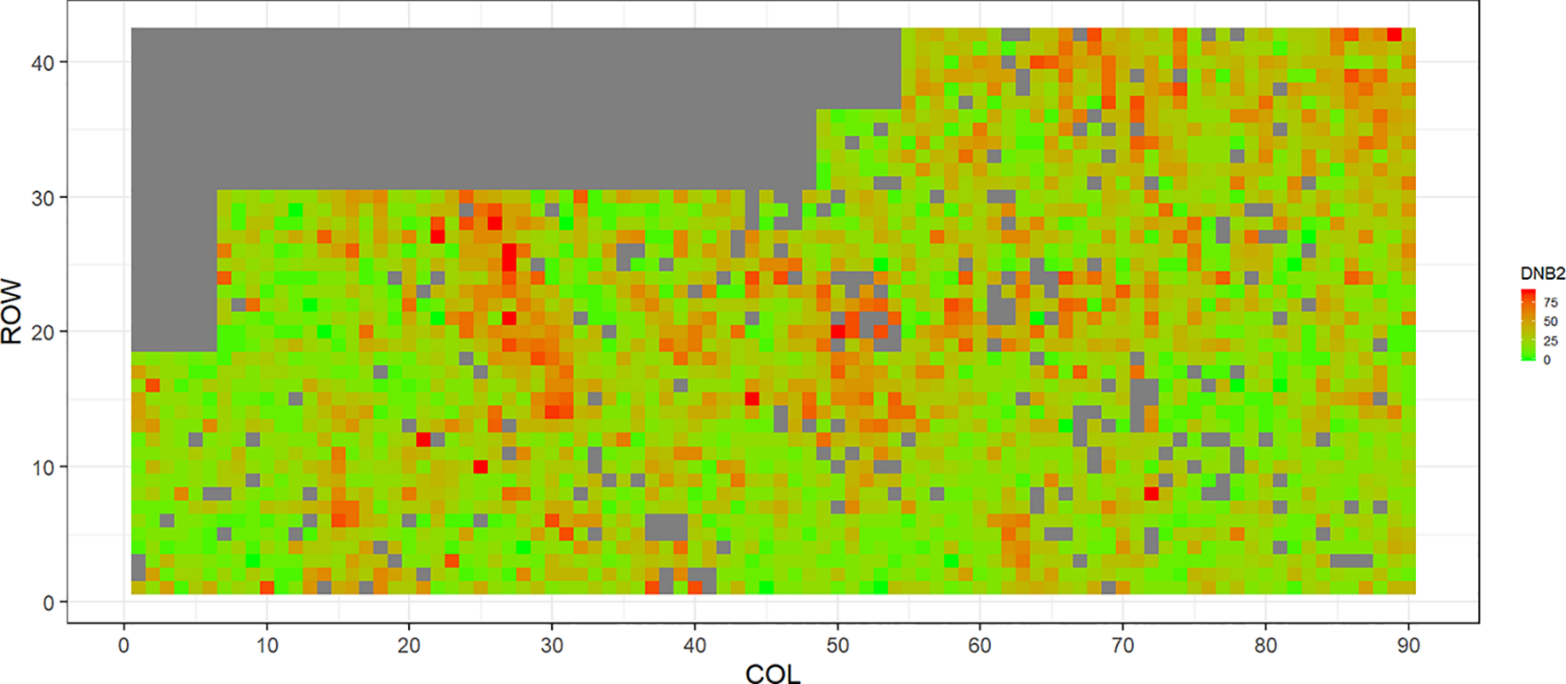
Spatial distribution of *Dothistroma* needle blight damage in Kinleith-D (DNB2). Smaller scores indicate low disease expression (greener color) and larger scores indicate high disease expression (redder color).

**Figure 4:**
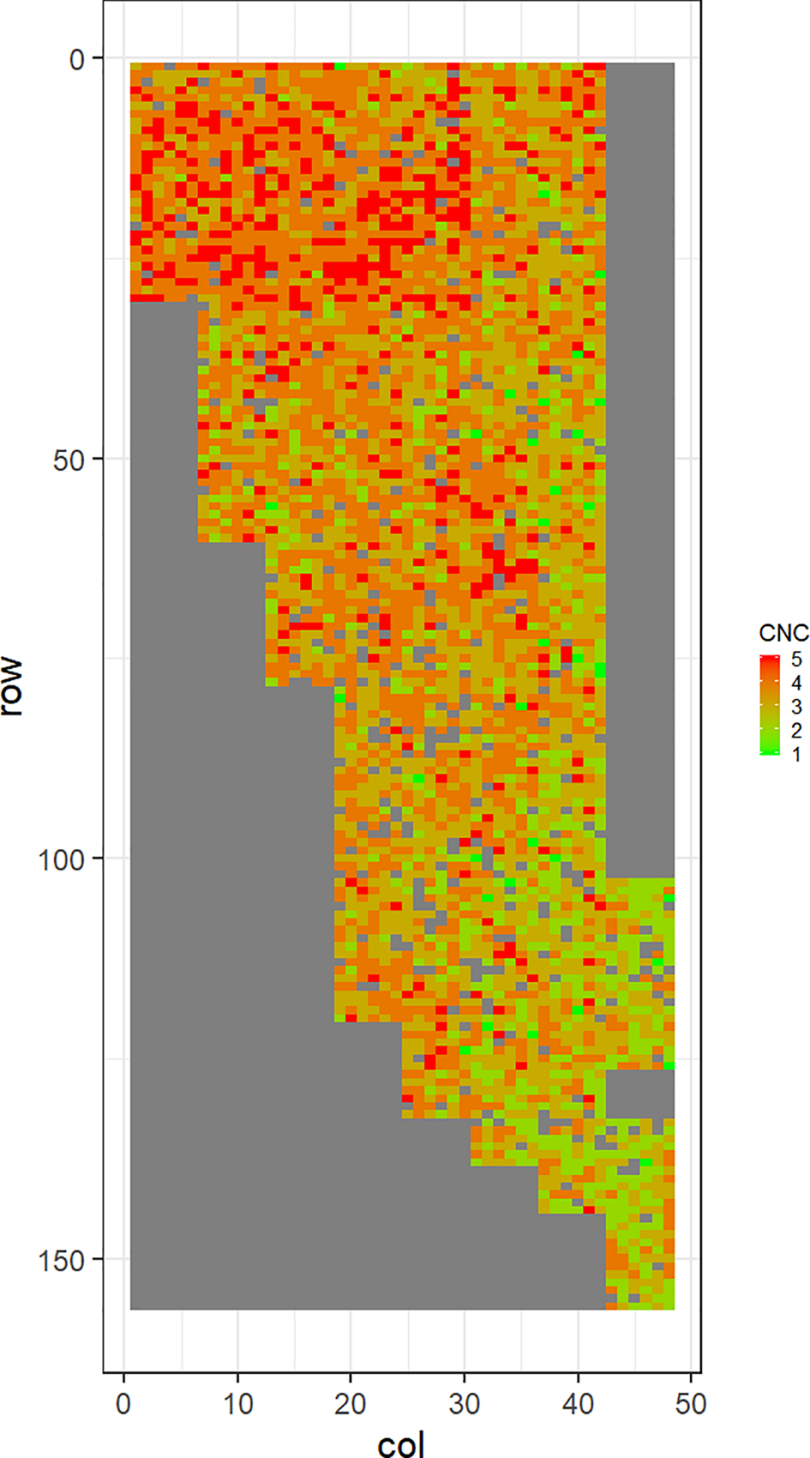
Spatial distribution of *Cyclaneusma* needle cast damage in Kinleith-C (CNC). Smaller scores indicate low disease expression (greener color) and larger scores indicate high disease expression (redder color).

**Figure 5:**
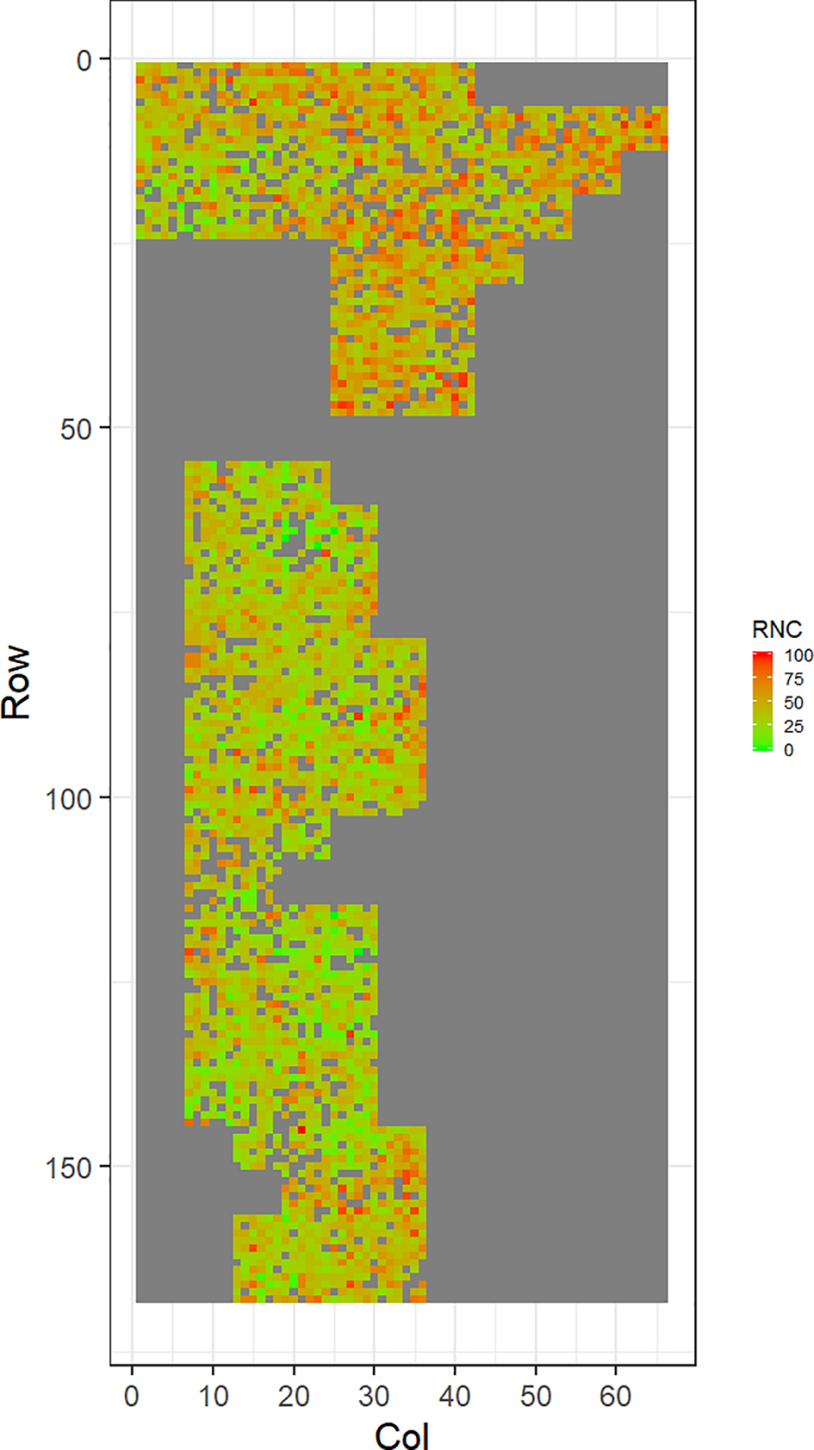
Spatial distribution of red needle cast damage in Mahurangi (RNC). Smaller scores indicate low disease expression (greener color) and larger scores indicate high disease expression (redder color).

**Figures 2**–**5** show the spatial distribution of the phenotypic expression for each trait in each site of the experiment, with each square representing an individual tree within the row/column design of each trial. DNB2 showed more clusters of infection compared to DNB1, with patches of infection mainly in the middle of the trial and fewer patches toward the edges. Similarly, CNC showed patches of heavy infection in the middle of the trial. RNC, however, showed fewer patches of infection in the middle of the trial, and only slight patches of infection toward the edges. Preliminary analyses showed that the spatial autoregressive mixed models were significantly better than the non-spatial mixed models based on the log likelihood comparisons (chi-square test). The individual-tree responses for DNB1, DNB2, CNC, and RNC were moderately heritable, with narrow-sense heritability estimates of 0.32, 0.34, 0.48, and 0.28, respectively (**Table 3**). The broad-sense heritability estimate for DNB1 was 0.52 compared with 0.37 for DNB2. The spatial variance represented 26%, 44%, 24%, and 32% of the total residual variance for DNB1 and DNB2, for CNC, and for RNC, respectively.

**Table 3:** Estimates of variance components, narrow-sense heritability (h^2^) and broad-sense heritability (H^2^), with approximate standard errors in parentheses for disease resistance traits.

Parameter	^1^DNB1	^2^DNB2	^3^CNC	^4^RNC
Block variance $\left(\sigma_{B}^{2}\right)$	0.03 (0.02)	0.23 (0.07)	0.00 (0.000)	0.02 (0.02)
Replicate variance $\left(\sigma_{R}^{2}\right)$	––––	––––	0.01 (0.004)	0.02 (0.02)
Additive genetic variance $\left(\sigma_{a}^{2}\right)$	0.67 (0.18)	0.55 (0.15)	0.03 (0.004)	0.36 (0.06)
Non-additive genetic variance $\left(\sigma_{\text{N}}^{2}\right)$	0.41 (0.11)	0.05 (0.09)	––––	––––
Residual variance $\left(\sigma_{e}^{2}\right)$	1.02 (0.04)	1.03 (0.07)	0.03 (0.003)	0.90 (0.06)
Narrow-sense heritability (*h^2^*)	0.32 (0.07)	0.34 (0.08)	0.48 (0.070)	0.28 (0.05)
Broad-sense heritability (*H^2^*)	0.52 (0.03)	0.37 (0.04)	––––	––––

1 DNB1: Dothistroma needle blight in Kaingaroa forest.

2 DNB2: Dothistroma needle blight in Kinleith forest.

3 CNC: Cyclaneusma needle cast.

4 RNC: Red needle cast.

### Genetic Correlations Between Traits and Sites

The genetic correlation for DNB1 and DNB2 with CNC resistance was strong and favorable (0.80 ± 0.10, and 0.81 ± 0.10, respectively; **Table 4**). This strong genetic correlation may indicate that selection for resistance to one disease based on resistance to the other disease could be effective. This is further supported by the predicted indirect selection responses, where indirect selection for CNC based on DNB scores at age 2 years should still gain reasonable per-generation selection efficiencies of 67% compared to direct selection for this trait.

**Table 4:** Genetic correlations between disease resistance traits, with standard errors in parentheses.

Trait	DNB2	CNC	^4^RNC
^1^DNB1	0.93 (0.03)	0.81 (0.10)	-0.12 (0.20)
^2^DNB2		0.80 (0.10)	-0.29 (0.20)
^3^CNC			0.04 (0.20)

1 DNB1: Dothistroma needle blight in Kaingaroa forest.

2 DNB2: Dothistroma needle blight in Kinleith forest.

3 CNC: Cyclaneusma needle cast.

4 RNC: Red needle cast.

Genetic correlation estimates between DNB and RNC, and CNC and RNC were not significantly different from zero and very imprecise.

The estimated genetic correlation between DNB1 and DNB2 was very strong and close to one (0.93 ± 0.03) indicating little evidence of G × E for this trait between these two sites.

## Discussion

In the current study, assessments for resistance to three needle diseases were undertaken in four separate trials. With naturally occurring infections, disease incidence and severity of expression would be expected to vary between sites, age of trees, and years. This could be due to variation of climatic variables, sun exposure and microsite characteristics, as well as changing physiology of the trees. Severity of DNB in the current study was higher than that reported by Klápště et al. (2020), who reported DNB severity of 12.2 in a 2-year-old *P. radiata* breeding trial assessed in 2016, also in Kinleith forest in New Zealand. The same authors reported severities of 27.4 and 23.4 for 3- and 4-year-old assessments during years 2017 and 2018. Severity for CNC in our study was lower than that reported by Suontama et al. (2019), who reported serious severity of CNC (mean score of 3.63) in a 9-year-old *P. radiata* trial located in Mangatu, on the east coast of New Zealand's North Island, and assessed in 2015. Severity of RNC in our study was substantially higher than that reported by Dungey et al. (2014), who reported mild severity of RNC (mean score of 18.4) in a 9-year-old *P. radiata* trial in Wharerata, also on the east coast of New Zealand's North Island, and assessed in 2011.

The incidence and severity of disease within the same field experiment can be spatially structured and unequally distributed. For DNB and CNC, patches of more severe infection mainly existed in the middle of the trials, with fewer patches toward the edges. Similar patterns of infection have been reported by Klápště et al. (2020) in Kaingaroa and Kinleith forests in New Zealand during assessments for DNB infections in 2- to 4-year old trials. On the other hand, for RNC, there were fewer patches of severe infection in the middle of the trial, with only slight patches toward the edges, with the infection apparently scattered without any specific pattern within the trial. These spatial variations observed in disease incidence and severity Could be due to microsite effects that result in hydrological variation within sites. Even small variations in elevation within sites can affect the distribution of precipitation and localized humidity, which in turn can strongly affect the moisture levels, nutrient supply, and disease spread (Li et al., 2003; Li and Yang, 2004). This could explain the difference between the average DNB score we observed in this current study in Kaingaroa and Kinleith forests. It could also explain the significant percentage of the total residual variance in all trials that was attributed to the spatial component. Therefore, removing the spatial component of the residual variance led to significant improvements in heritability estimates, as has been reported in other studies (Dutkowski et al., 2002; Dutkowski et al., 2006).

Heritability estimates for DNB were in agreement with previous estimates in the literature, which range from 0.05 to 0.75 for *P. radiata* in New Zealand and Australia, and for Scots pine in the United Kingdom (Carson, 1989; Ivković et al., 2010; Perry et al., 2016). Heritability of CNC was also in broad agreement with other studies performed in New Zealand (King and Burdon, 1991; Kumar et al., 2008; Suontama et al., 2019). The heritability of RNC resistance was similar to the heritability estimates obtained previously for *P. radiata* in New Zealand (Dungey et al., 2014; Graham et al., 2018). These estimates of heritability indicate that a portion of the variation of disease resistance among genotypes was attributable to genetic variation and that breeding values could be predicted with reasonable accuracy to enable selection for resistant genotypes.

The economic impact of needle diseases mainly results from the reduction in growth rate of trees, although needles of different ages in the perennial foliage of pines do not have equal contribution to the growth of trees. Rook et al. (1976) reported that defoliation of 1-year-old needles reduced height, basal area, and volume growth of *P. radiata* by 53%, 73%, and 77%, respectively. Applying these numbers to our study in terms of potential growth loss due to CNC-induced defoliation we might expect 76% of the trees (scores 3 and 4) to lose up to 5% of height, 16% of basal area, and 18% of volume growth. Additionally, 13.5% of the trees (score 1 and 2) might be expected to lose up to 14% of basal area. Furthermore, 10.5% of the trees that suffered from severe defoliation (score 5) could lose up to 26% of height, 63% of basal area, and 70% volume growth. For DNB, the disease causes large losses in growth relative to the proportion of affected crown. For example, Van Der Pas (1981) reported a reduction of height, diameter, and volume of final crop by 0.29%, 0.47%, and 1.00%, respectively, for each percentage increase in average disease level. In our study, this loss would be translated into loss of height by 6.2–8.6%, loss of diameter by 10–14%, and volume loss by 20.5–28% for DNB1 and DNB2 trials.

Genetic correlation between DNB1 and DNB2 was close to one, indicating the absence of G×E interaction for DNB resistance in the two progeny trial sites. This indicates that families were ranked consistently between the two sites. The lack of a significant G × E interaction for DNB resistance could be explained by low genetic diversity in the *Dothistroma septosporum* population in New Zealand due to absence of the teleomorph (sexual form) *Mycosphaerella pini* E. Rostrup apud Monk (Hirst et al., 1999). Only one strain has been isolated from New Zealand since the disease was first introduced (Hirst et al., 1999).

The genetic correlation between needle damage due to DNB and needle retention in CNC infected trees was both favorable and strong, suggesting that selection for families with resistance to both DNB and CNC is possible, and could lead to a simplified selection for multiple disease resistant genotypes, by selecting genotypes for resistance to CNC based on DNB scores. However, the exact biological mechanisms underlying this genetic correlation are not known. We speculated that this correlation might be related to the taxonomic similarities between the causative agents for DNB and CNC. Both pathogens are under the same division (Ascomycota) of the Fungi kingdom, and both use similar mechanisms to infect the needles (Bulman and Gadgil, 2001; Bulman et al., 2004; Watt et al., 2012; Bulman and Gardner, 2014). One key difference between these pathogens is the location of and agents for spore dispersal: *Dothistroma* spores are released from fruiting bodies in live and dead needles and dispersed by water splash or wind through misty conditions, while *Cyclaneusma* spores are released from live and dead needles on the forest floor and dispersed by wind in misty conditions (Brendel et al., 2002; Watt et al., 2012). The non-significance of genetic correlations between DNB or CNC and RNC could be explained by the weak level of genetic relatedness (i.e., low number of shared parents) between genetic trials. For example, only 20% of parents were shared between DNB and RNC trials compared to 87% of parents shared between DNB and CNC trials. Another possible explanation might be related to taxonomic differences between RNC (an oomycete) and DNB or CNC (both fungi).

Earlier studies have reported mixed results on the potential of indirect selection to improve resistance against multiple diseases. For example, moderate to high genetic correlations found between resistance to diseases caused by three *Fusarium* species indicated that selection for resistance to root rot caused by a specific *Fusarium* species conferred a general resistance to rot caused by all three species (Miller-Garvin and Viands, 1994). On the other hand, indirect selection failed to improve multiple disease resistance in *Vigna radiata,* mungbean; a lack of genetic correlations among resistance to bacterial leaf spot, yellow mosaic, and cercospora leaf spot led the authors to conclude that resistance to each disease was inherited independently (Thakur et al., 1977). Further studies to investigate seasonality and environmental conditions, as well as improved connectedness among traits, would enable a deeper knowledge of the genetic background of disease resistance traits in the present study.

The findings of this study must be viewed in light of some inherent limitations. First, families were not fully replicated on all sites. Second, the study relies on natural infections thus disease expression may be confounded with different site environments and different tree ages among the sites. Third, potential bias is hard to avoid due to the inherent subjectivity of visual disease assessments performed by human assessors, with factors such as light conditions, time of day, and weather conditions impacting on the assessor's accuracy. Fourth, we are not able to compare the results of genetic correlations across studies, mainly because variance components including genetic and non-genetic variances, heritability estimates and correlations between traits will change as a function of environmental conditions (i.e., temperature, humidity, light exposure), age of trees, and different levels of disease infestation rate (De La Mata et al., 2017). For example, Klápště et al. (2020) reported a decrease of heritability estimates and accuracy of estimated breeding values of DNB in *P. radiata* in New Zealand when the disease assessment was performed under sub-optimal weather conditions or when the trees are still too young to allow for reliable phenotyping. One possible solution to reduce this bias is to perform the assessments under optimal age and environmental conditions for disease expression (e.g., assessment for CNC in an older trial). In addition, precise identification of the pathogen causing the needle damage through laboratory testing on a random subset of trees should ideally be conducted prior to full-trial visual assessments. Furthermore, the accuracy of disease assessments and the comparative ability of all disease assessors should routinely be tested at least annually, prior to any assessments (Bulman et al., 2004). Despite these limitations, we were still able to detect a significant genetic correlation between DNB and CNC.

The current study also shows the potential of selecting genotypes with resistance to multiple diseases using estimated breeding values (EBV). The main advantage of the multi-trait model over a single-trait model is that additional information from genetically correlated traits can be used to achieve higher EBV accuracies. In a simulation study, the accuracy of EBVs increased by 25% when changing from a single-trait to a multi-trait model, where the genetic correlation between the simulated traits was set at 0.5 (Guo et al., 2014). This is especially valuable in cases where the traits of interest have low heritability, or are expressed late, or are costly and difficult to measure, as is the case for CNC where disease expression does not usually become obvious in stands less than 6 years of age (Bulman and Gadgil, 2001), making it hard to assess due to the height of tree crowns and canopy closure.

Our study showed that selection for resistance to CNC based on the DNB scores could still gain a reasonable per-generation selection efficiency of 67%, compared with direct selection for CNC, with the added benefit of significant cost reductions by reducing the number of disease assessments required. Furthermore, the availability of multi disease-resistant selections will decrease the need for chemical treatments against pathogen infection, which are often economically unfeasible and largely unsuccessful for CNC (Vanner et al., 1986). To have any impact, chemical treatments have to be applied three or four times per season, the cost of which is often not compensated for by the extra growth and volume produced (Bulman and Gardner, 2014). However, we need to be mindful that the assessment of CNC resistance has been done at an early age, and the effectiveness of indirect selection for CNC resistance based on DNB scores would rely on strong genetic correlations between early and later assessments for CNC resistance. Therefore, further studies are required to investigate the correlation between early resistance for CNC at age 5 and CNC resistance at age 11-20. More data would allow for more reliable estimates of the genetic correlations between resistances to the diseases evaluated, especially between RNC and the other two diseases. Additional studies are required to understand the biological mechanisms for resistance to each disease, and how these pathogens interact, and the mechanisms that impart host resistance, and interact among different genotypes.

## Conclusions

Resistance to needle damage due to DNB, CNC, and RNC, is moderately heritable, indicating that selection for improved resistance to each of the three diseases is possible. The genetic correlation between the response to DNB infection and the early resistance for CNC was high and favorable, suggesting that selection for genotypes with apparent multiple disease resistance, i.e multiple similar additive genetic effects on both traits, may be possible to facilitate selection for disease resistance in the future breeding program for *P. radiata* in New Zealand.

## Data Availability Statement

The datasets presented in this study can be found in online repositories. The names of the repository/repositories and accession number(s) can be found below: https://doi.org/10.5281/zenodo.3354594.

## Author Contributions

AI analyzed the data and wrote the manuscript. ET and HD conceived and supervised the study, made substantial contributions to the interpretation of the results, and contributed to revision the manuscript. SK, MS, JK, and NG made substantial contributions to the interpretation of the results and contributed to revision the manuscript. All authors contributed to the article and approved the submitted version.

## Funding

The study was funded by the Radiata Pine Breeding Company and NZ Ministry of Business, Innovation and Employment (MBIE) joint project RPBC1301, Specialty Wood Products Research Partnership Program (SWP) contract nr. C04X1104, and MBIE Strategic Science Investment Fund contract nr. C04X1703.

## Conflict of Interest

Authors AI, MS, JK, SK, NG, ET and HD were employed by Scion, New Zealand.

The authors declare that this study received funding from NZ Ministry of Business, Innovation and Employment and the Radiata Pine Breeding Company. The Radiata Pine Breeding Company. was involved in phenotypic data collection. The funders were not involved in the study design, analysis, interpretation of data, the writing of this article or the decision to submit it for publication.
